# Effectiveness of group work among the final radiography students of the National Diploma

**DOI:** 10.4102/hsag.v29i0.2681

**Published:** 2024-09-27

**Authors:** Kealeboga P. Menwe, Lynne Hazell, Heather A. Lawrence

**Affiliations:** 1 Department of Medical Imaging and Radiation Science, Faculty of Health Science, University of Johannesburg, Johannesburg, South Africa; 2 Department of Radiography, Faculty of Health Sciences, University of Johannesburg, Johannesburg, South Africa

**Keywords:** group work, radiography, effectiveness, students, experiences

## Abstract

**Background:**

Group work is employed in higher education institutions to foster communication, collaborative learning, leadership qualities and teamwork skills. The rationale behind group work as a learning strategy is to ensure that graduates are equipped with teamwork skills.

**Aim:**

The aim of this study was to establish whether the final-year radiography students of the National Diploma curriculum in three universities in South Africa were exposed to effective group work.

**Setting:**

The study was conducted in three universities in South Africa, all of which offer diagnostic radiography programmes.

**Methods:**

This study used a non-experimental descriptive design to collect data. A purposive sampling technique was used to select participants. This was an attempt to conduct a census on 167 students, out of which 124 students responded to the questionnaire, giving a response rate of 74.25%.

**Results:**

The findings show that the majority (*n* = 81; 66.9%) of students did not enjoy group work, as 101 (81.5%) of them preferred less group projects. The results further demonstrated that participants rated factors such as co-operation, leadership, choice, diversity and effectiveness of group work neutral on the Likert scale.

**Conclusion:**

The study results imply that factors that contribute towards the effectiveness of group work should be incorporated into group activities in order for student radiographers to be exposed to effective group work. The gaps identified in this study indicate the need for a follow-up study within the current 4-year bachelor’s degree in radiography.

**Contribution:**

Identification of a gap in radiography education, which group work needs to be facilitated effectively.

## Introduction

### Background and literature review

Healthcare systems are complex, open to change and difficult to predict; as the environment is constantly evolving, new problems can emerge. Therefore, effective teamwork among healthcare professionals is required to adapt to these challenges (Anderson, Lavelle & Reedy 2021; Botha & Sebelego 2022). Teamwork appears to be a challenge among the healthcare professionals, as Anderson et al. (2021) indicate that teamwork in healthcare is difficult because team members are from different professional backgrounds. Botha and Sebelego (2022) are of the view that healthcare professionals have little exposure to other healthcare professions because they are educated in silos; hence poor collaboration can be the result. In the nursing discipline, clinical staff indicated that nurses were lacking in communication and collaboration skills in the workplace (Yeung et al. 2023). Likewise, radiographers lack knowledge on interprofessional communication and collaboration (Fatahi, Kustrimovic & Elden 2020; Lundvall, Dahlström & Dahlgren 2021). Stadick (2020) argues that most of the patients die in hospitals from medical errors because of poor interprofessional communication and collaboration. As a result, the World Health Organization (WHO) demanded that healthcare professional students be taught to work together to deliver safe and effective patient-centred care. It means that healthcare professionals’ educators face an enormous challenge in preparing students to deal with the demands of the rapid changes in the healthcare systems (Yeung et al. 2023). Therefore, there was a need to teach radiography students to function optimally in a healthcare team as it is crucial to address the current complex healthcare needs being experienced worldwide (Botha & Sebelego 2022). Literature suggests that real-world experience could be enhanced by group work activities that are taught at the university (Cartwright et al. 2021; Hung & Mai 2020; Menwe 2019). Group work could be employed to foster teamwork skills and effective communication. For the purpose of this study, effective group work will be defined as a process where a number of radiography students learn more effectively together by sharing knowledge, ideas and experiences to develop the skills of knowledge sharing, communication, teamwork, collaboration and critical thinking (Menwe 2019).

At the time of the study in 2015, most universities were offering 3-year National Diploma (NQF) exit level 6 radiography programme. The final radiography students of the old curriculum were being prepared for community service for the subsequent year. At the same time, universities offering radiography programme were moving away from a 3-year National Diploma to a 4-year bachelor’s degree in radiography (Pieterse, Lawrence & Friedrich-Nel 2016). The learning outcomes of the bachelor’s degree aim to produce students who can perform professional duties with confidence in collaboration with other healthcare professionals to promote efficient and effective delivery in the radiography profession and healthcare service in general (Pieterse et al. 2016). In addition, students must communicate effectively in both the learning and healthcare environments and demonstrate skills to share information with other healthcare workers to deliver quality patient care (South African Qualifications Authority [SAQA] 2001). The stated learning outcomes are crucial for radiography students. Hence, the radiography curriculum requires them to be placed in a training centre accredited by the Health Professions Council of South Africa (HPCSA) so that they can apply their theoretical learning in clinical practice (Hazell, Lawrence & Friedrich-Nel 2020:e238). Prior to the transition, the effectiveness of group work among the final-year radiography students of the old curriculum had not been assessed and was an unknown factor (Pieterse et al. 2016). Therefore, a study was warranted within the diploma-driven environment to afford the universities offering radiography programmes the opportunity to determine whether group work was effective in their curriculum so that interventions could be implemented in the new bachelor’s degree programme if it was found that group work was not facilitated effectively (Pieterse et al. 2016).

Radiography students are required to meet specific outcomes prescribed by SAQA before they qualify; hence the radiography curriculum incorporates group work as one of the critical cross-field outcomes (Van de venter & Engels-Hills 2022; SAQA 2001). Group work as a teaching approach in the tertiary classroom has been explored by several studies, and much has been written about its benefits. Qu and Cross (2024) state that a well-designed group task encourages and motivates learners by offering options of varied tasks and grouping structures that help to promote self-regulation, autonomy, sustained effort, persistence and interest. Employing group work as a teaching strategy promotes student collaboration, the development of key graduate employability skills, problem solving, communication and teamwork skills (Hall, Erasmus & Haywood 2022; Hung & Mai 2020:445; Seithers, Amankulova & Johnstone 2022). The rationale behind group work as a learning strategy is to ensure that graduates are work ready, as employers often express their requirements in terms of skills rather than subject knowledge, and they prefer a person who can work well with others (Cartwright et al. 2021; Hall et al. 2022). Situmorang (2021) conducted a study on group work, and the findings showed that a majority of students (61.34%) strongly agreed that group work provided them with opportunities to share ideas with their peers. In terms of improving communication, group work allows students to negotiate to control input, seeking information and conformation and keeping a conversation going on in speaking activities (Hung & Mai 2020). The study by Chiriac (2011) on ‘Why group work?’ highlights developmental and motivational approaches to clarify how learning is improved through group work. The former is based on Piaget and Vygotsky’s assumption about learning ensuing from intrinsic processes and development, and the latter emphasises extrinsic rewards. According to the developmental approach, group work improves learning through interactions between students, discussions in the group and the ability to share information regarding solution strategies (Chiriac 2011). Although several studies have shown the advantages of group work, it comes with challenges such as group dynamics complexity, tension over completing tasks against a deadline, students lacking the necessary collaboration skills, unequal individual participation in group tasks that can be demotivating and social loafing (Hall et al. 2022; Qu & Cross 2024). Diversity in terms of personality, cognition, biological sex, race and ethnicity can impact groups negatively or positively. When it is positive, students’ experience increased innovative and creative ideas, while if negative it could lead to increased conflict and lower cohesion (Samadi et al. 2022). Social loafing occurs when the bulk of work usually falls to one or two individuals and the rest of the group is seen to ‘piggyback’ on these students (Chiriac 2011; Hall et al. 2022; Situmorang 2021; Thom 2020). Hence, it is the responsibility of the educator to give the necessary guidance to ensure that the group functions effectively (Frey, Fisher & Everlove 2009; Hall et al. 2022). Chiriac (2011) points out that educators are reluctant to use the group work method in the classroom because they are used to traditional teaching methods, while Qu and Cross (2024) are of the view that educators could be struggling to structure and design group tasks and class time management. Therefore, the literature suggests that group work be structured (Brannen et al. 2021; Cartwright et al. 2021). For the purpose of this discussion, the structural considerations for effective group work will be discussed using the following key structural considerations: co-operation, assertiveness, responsibility, autonomy, communication, co-ordination, mutual trust and respect (Menwe 2019).

### Aim of the study

The aim of this study was to establish whether the final radiography students of the National Diploma curriculum in three universities in South Africa were exposed to effective group work.

## Research methods and design

### Design

A non-experimental descriptive design was employed to collect quantitative data. The study design was selected to describe the group work experiences of students. A self-developed questionnaire was designed to collect data from student diagnostic radiographers, as no previously published studies to address the group work experiences of student radiographers could be identified. The questionnaire was divided into sections A, B and C. Section A comprised nine questions that focussed on acquiring demographic and general information. Section B comprised 33 closed-ended questions that were designed to measure the 7 elements of group work identified in the literature (Menwe 2019), namely co-operation, assertiveness, responsibility, autonomy, communication, coordination and mutual trust and respect. These elements were adapted from the work of Gagnon and Roberge (2012). Section C comprised four open-ended questions providing the participants with the opportunity to describe their group work learning experiences in their own words. The questionnaire was in a hardcopy format so that participants could complete it manually.

### Setting

The study was conducted in three universities in South Africa that offered the 3-year National Diploma in radiography.

### Study population and sampling strategy

The data were collected from April 2015 until July 2015 because students were on clinical block and some universities were on recess at the time. The population for this study included the final diagnostic radiography students of the old curriculum who were registered for undergraduate diagnostic radiography in 2015, at three universities in South Africa (*n* = 167). A census of this population was attempted. A response rate of 74.25% was achieved with 124 students returning a completed questionnaire. These students were purposely selected to determine their experience in group work as they have already been exposed to group work in previous years. For the purpose of this study, 3 years of experience in group work were deemed sufficient as students were exposed to group work throughout the programme. The final radiography students of the old curriculum were chosen as the participants in the study because they had more group work experience. The first-year students were excluded because they did not have any group work experience at the time of data collection. They had been on campus for only 2 months. The second-year groups were excluded because they had only 1 year of group work experience, which was not sufficient for this study. From 124 participants, University 1 comprised 58 participants (46.8%), University 2 comprised 33 participants (26%) and University 3 comprised 33 participants (26%) (Menwe 2019).

### Data collection

The data collection process began once approval, and ethical clearance for the study was obtained from the Faculty of Health Science Higher Degrees Committee and the Research Ethics Committee at the University of Johannesburg. Ethical clearances from the two other universities were also given following the University of Johannesburg. Arrangements were made with these three universities for data to be collected. Arrangements were made with these three universities for data to be collected based on the availability of the students. Because of the clinical block system, the researcher had to collect data after students completed their clinical blocks at their respective training sites. Therefore, data were collected on different dates. In University 1, the researcher arranged with an academic staff member from a different discipline in the same faculty as radiography to facilitate the data collection process, and data were collected on 24 April 2015. In University 2, the researcher personally delivered and collected completed questionnaires on 23 July 2015, and the researcher had no relationship with participants at all. For University 3, an academic staff member who collected data had no relationship with the participants as well, and the data were collected on 20 July 2015. In all the universities where the questionnaire was completed, the participants were recruited to complete the survey in one room at the same time. Participants were provided with a consent form before they completed the questionnaire, which stated the purpose of the study and clearly indicated that participation was voluntary. All participants were invited to participate without coercion. There were no anticipated risks for participants involved in the study. The researcher and the academic staff members explained the study aim to participants prior to data collection. To maintain confidentiality, the universities were identified as University 1, University 2 and University 3. Moreover, the questionnaires were completed anonymously as no names were assigned to the data collection tool. After the completion of the survey, the questionnaire was inserted into a sealed box with a slit on the top. The questionnaires were colour coded according to university in order to differentiate between the three universities for data capturing purposes. The colour codes and the corresponding university were known only to the researcher. As there was no record of previously published studies to address the group work experiences of student radiographers, a self-developed questionnaire was developed to collect data from the participants. After an extensive literature search, questions were developed from a variety of sources, as indicated within brackets (Analoui, Sambrook & Doloriert 2014; Gagnon & Roberge 2012; Grizmek, Mark & Kinnamon 2014; Liu & Dall’Alba 2012; Marks & O’Connor 2013; Retna 2015; Smith & Rogers 2014; Swaray 2012). The literature search identified seven essential elements of group work that were integrated into the questionnaire design to validate the tool. Questions incorporated the collaborative essential elements for successful group work as described by Gagnon and Roberge (2012). Each element was measured by three questions. More questions were developed by adapting questions from the literature. The questionnaire was peer reviewed by radiography educators at a university in South Africa who formed a focus group to ensure face and content validity. Amendments were made to the questionnaire after it was critiqued by radiography educators. Before the questionnaire could be administered to the participants, it was piloted with a small sample (*n* = 24) of second-year radiography students. Further amendments were made after the results of the pilot study. For reliability, the overall Cronbach’s alpha coefficient of the tool was 0.848.

### Data analysis

The quantitative data were generated by analysing the participants’ responses to the Likert scale using the Statistical Package for the Social Sciences (SPSS) programme to generate means and standard deviations for the scores achieved. Tables are used to display the results, and a level of statistical significance of *p* < 0.05 was used in this study to compare variables (Pallant 2013). In addition, verbatim quotes were used to provide additional insight into the quantitative results. Based on the number of items in the questionnaire, Bonnette formula was used for Equation 1:


$$\left[\left\{\left(\frac{2k}{k-1}\right)\left(z_{\alpha /2}+z_{\beta }\right)\right\}/ln{\left(\delta \right)}^{2}\right]+2$$ [Eqn 1]

Where Equation 2 is:


$$\delta =\frac{1-CA_{0}}{1-CA_{1}}$$ [Eqn 2]

*k* = number of items (likert scale) in questionnaire

*CA*_0_ = The value of Cronbach’s alpha at null hypothesis

*CA*_1_ = The expected value of Cronbach’s alpha

The sample size was calculated on a power of 0.80 and an expected power of 0.866 (*Power = 1 - β*) while the probability of a type I error (*α*) was set at 0.05.

The questionnaire had 31 items, of which the reliability of its measurements needed to be measured (CA0 and CA1 were identified at 0.84 and 0.866, respectively). Power was set at 80%, and the alpha value was set at 0.05.

The minimum sample size required based on formulas: (1) and (2) represents the relative change in non reliability (error variance) between the Cronbach’s alpha under the null hypothesis and the Cronbach’s sample size is shown as Equation 3

Calculations:

*α* = 0.05

*β* = 0.2

*k* = 15

*CA*_0_ = 0.84

*CA*_1_ =0.866


$$\begin{array}{l} \delta =\frac{1-0.8}{1-0.866}=1.493\\ n=\left[\frac{\left\{\left(\frac{2(15)}{15-1}\right){\left(z_{0.025}=1.96+z_{0.2}=0.84\right)}^{2}\right\}}{\ln{\left(1.493\right)}^{2}}\right]+2\\ =\frac{16.203}{0.160}+2\\ =103.025\\ \approx 104 \end{array}$$ [Eqn 3]

Descriptive analysis was used to analyse the survey results, including counts, percentages and standard deviations (Pallant 2013) using IBM SPSS version 22. These summary statistics were used to describe the sample and the distribution of the responses to the individual questions about group work. The adequacy of the dataset for factor analysis was confirmed through the Kaiser–Meyer–Olkin (KMO) measure and Bartlett’s test of sphericity (see Table 1). The KMO measure was 0.810, indicating a high level of sampling adequacy for the dataset. A KMO value closer to 1.0 suggests that the variables share common factors, which is ideal for factor analysis. Bartlett’s test produced an approximate chi-square value of 863.857 with 190 degrees of freedom and a *p*-value < 0.001. This highly significant result indicates that the correlation matrix is not an identity matrix, justifying the use of factor analysis. The significant *p*-value (less than 0.05) suggests that there are significant correlations among the variables, validating the suitability of the data for factor analysis.

**TABLE 1:** Kaiser–Meyer–Olkin and Bartlett’s test.

Test	Variable	Value
Kaiser–Meyer–Olkin measure of sampling adequacy	-	0.8
Bartlett’s test of sphericity	Approx. Chi-square	863.9
	*df*	190.0
	Sig.	0.0

Sig., significance; *df*, degrees of freedom; Approx., approximately.

Exploratory factor analysis (EFA) was conducted to explore the structure of the theoretical constructs using principal axis factoring with Oblimin rotation. The dataset comprised 124 valid cases, with no exclusions based on listwise deletion across all procedures. Detailed item statistics and corrected item-total correlations were computed for each theoretical factor, revealing insights into item contributions and potential areas for scale refinement. The exploratory factor analysis led to the production of five factors. To finalise the factors, items with a loading < 0.400 were removed because factor loading was low. As a result, four factors remained. For clarity, these factors contribute towards the effectiveness of group work and can be described as follows:

- Factor 1: co-operation in group work;
- Factor 2: leadership in group work;
- Factor 3: choice in group work; and
- Factor 4: diversity in group work.

Means and standard deviations for the factors were calculated and presented. Table 2 presents the means and standard deviations of the empirical factors contributing to effective group work.

**TABLE 2:** Empirical factors contributing to effectiveness of group work (*N* = 124).

Factors contributing to effectiveness of group work	Mean	Std. deviation	Min	Max
Co-operation in group work	3.05	0.84	1.17	5.00
Leadership of group work	3.44	0.71	1.00	5.00
Choice in group work	2.91	0.94	1.00	5.00
Diversity of group	3.41	1.00	1.00	5.00
Effectiveness of group work	3.37	0.71	1.08	4.83

Std., standard; Min., minimum; Max., maximum.

The higher mean score suggests the factor’s tendency to be present within group work. However, as can be seen in Table 2, the mean scores of the set of entire empirical factors are close to neutral on the Likert scale. The internal consistency of these new factors was tested using Cronbach’s alpha: co-operation = 0.840, leadership = 0.693, choice = 0.478 and diversity = 0.569. Factors were computed based on the exploratory factor analysis results by calculating the mean of items that contribute to each factor. The relationships between factors and effectiveness were examined using Pearson’s correlation. According to O’Dwyer and Bernauer (2014), Pearson’s correlation is a single numerical value between −1 and +1 used to indicate the strength and direction of the relationship between variables. A comparison of factors by predictor variables such as university and biological sex of the respondent was conducted using the independent samples t-test (biological sex) and one-way ANOVA (university). For the non-parametric test, the Kruskal–Wallis test was employed for the comparison of scores between the three universities, and the Mann–Whitney test was employed for a comparison between two variables (Pallant 2013). The level of statistical significance of *p* < 0.05 was used in this study. The Kolmogorov–Smirnov test was used to assess the normality of the distribution of scores, and Lavene’s test was used to compare the mean scores between two different variables (Pallant 2013:63, 241). The assumption of normality was assessed at the *p* < 0.05 level in this study (Pallant 2013).

**FIGURE 1:**
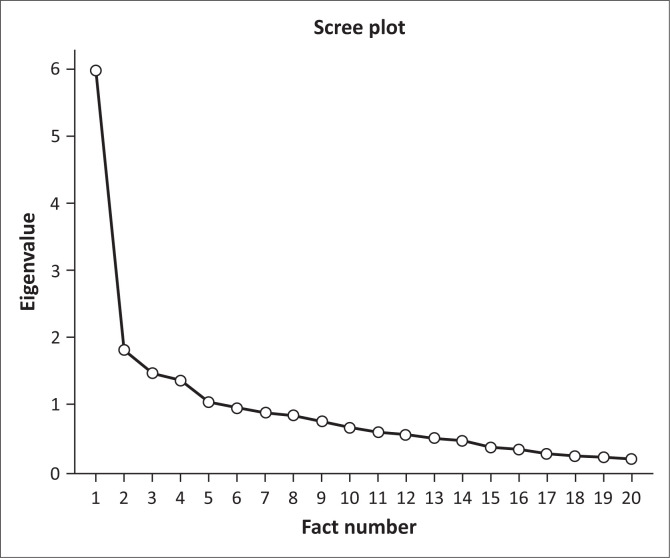
Scree test of four factors.

### Ethical considerations

Ethical clearance to conduct this study was obtained from the University of Johannesburg Research Committee (Registration no: REC-241112-035). This study followed all the ethical standards required when human subjects are used in the research project. After the data collection process, the researcher locked all the information acquired from the participants in a safe place where access is only limited to the researcher for 5 years. There was no direct benefit to the participants. Moreover, the results of the study will be used to add knowledge to radiography education.

## Results

For descriptive statistics, data were analysed to determine whether students enjoy group work, would prefer to have fewer group projects, perceived that the lecturers adequately explained the outcomes that should be achieved through the group work activity and perceived that the lecturer effectively facilitated group work. The results are presented in Table 3.

**TABLE 3:** Descriptive statistics results.

Questions	Description	Frequency (*n*)	Percentage (%)
Enjoyment of group work	Yes	40	33.1
	No	81	66.9
Indication of a preference for fewer group projects	Yes	101	81.5
	No	23	18.5
Students’ perceptions of group work outcomes adequately provided by the lecturer	Yes	117	94
	No	7	5.6
Participants’ perception of facilitation of group work by the lecturer	Agree	101	81.5
	Disagree	22	17.7

The inferential statistics below report on the significant results that were found between the empirical factors, such as co-operation, leadership, diversity and choice and variables, such as biological sex, enjoyment of group work, facilitation and outcome of group work. The relationship between the empirical factors and effectiveness was examined using Pearson’s correlation. Pearson’s correlation coefficient was calculated to explore the strength of the relationship between the empirical factors and effectiveness. The *r*-value indicates the strength of the correlation and a *p*-value of less than 0.05 indicates a significant correlation (Pallant 2013:134). The Mann–Whitney test was used to test the differences between the empirical factors and variables. The results show that the rating of empirical factors and the effectiveness of group work differ by university, biological sex, enjoyment of group work and preference for fewer group projects, while other variables do not seem to influence the rating of effectiveness. The Kruskal–Wallis test was used to compare the rating of co-operation, leadership, choice and diversity among three universities.

### Co-operation

The Mann–Whitney test for the correlation of biological sex, enjoyment of group work and preference to do less group projects significantly impacts on the facilitation of group work with co-operation. The results indicate that male participants rated co-operation higher than female participants (*p* = 0.016). The participants who enjoy group work rated co-operation higher than participants who did not enjoy group work (*p* = 0.000). The participants who prefer to do less group projects rated co-operation lower than participants who enjoy group work (*p* = 0.001). Lastly, participants who agree that the lecturer facilitated effective group work rated co-operation higher than participants who disagreed (*p* = 0.002). The Kruskal–Wallis test for comparison between the three universities and co-operation showed a strong correlation between co-operation (*r* = 0.625, *p* = 0.000) and the participants’ overall rating of the effectiveness of group work projects. The significant positive correlation observed indicates a higher rating of co-operation, which promotes the effectiveness of group work.

#### Verbatim quotes

The verbatim quotes supporting the requirement for the element of co-operation are as follows:

‘Group work is not always easy as some group members are not always hardworking or cooperative as expected.’ (Participant 38, Student, 19–35 years)

‘Group work has created more stress for me because group members do not work at the same level and some group members do not give their best and it brings the group marks down.’ (Participant 46, Student, 19–35 years)

‘Challenging at time as other members of the group turn to take over. I feel sometimes I did not contribute to the best at my ability.’ (Participant 60, Student, 19–35 years)

‘I don’t like group work. It is time consuming when you have to wait for other members of the group to submit their work. When work is divided members only concentrate on their parts of the work.’ (Participant 109, Student, 19–35 years)

‘No Trust especially with people you don’t know and people never stick to deadlines, tend to take advantage of 1 or 2 members in group. When we are given a task and asked to complete in a group, we chat.’ (Participant 124, Student, 19–35 years)

### Leadership

The Mann–Whitney test for the correlation of biological sex with leadership indicates that there is a significant relationship between biological sex and leadership (*p* = 0.014). Female participants rated leadership higher than males. The Kruskal–Wallis test for comparison among the three universities and leadership shows a significant difference among the three universities and leadership (*p* = 0.033). One university student rated leadership higher than the other; perhaps this could be because of the requirements of their projects that emphasised good leadership. The Kruskal–Wallis test for comparison among the three universities and leadership shows a moderate correlation between leadership of group work and effectiveness (*r* = 0.320, *p* = 0.000). This means that leadership could be incorporated into the learning outcomes for group projects to promote effective group work.

#### Verbatim quotes

The verbatim quotes regarding the role of the leader in group work are as follows:

‘I find if I’m the group leader, I do most of the work, collecting the information and giving it to the group, putting the assignment together.’ (Participant 93, Student, 19–35 years)

‘Some students always want to be group leader therefore others are not granted this opportunity like stated before not everybody does their bit and people prefer to rely on others to get work done.’ (Participant 26, Student, 19–35 years)

‘Yes, in some group activity we don’t elect leaders but automatically someone becomes in charge and it becomes a problem for some students.’ (Participant 91, Student, 19–35 years)

### Choice

The Mann–Whitney test for comparison between biological sex and choice indicates that biological sex significantly impacts on choice (*p* = 0.006); male participants rated choice higher than female participants. The Kruskal–Wallis test for comparison between universities and choice shows a significant correlation between choice in group work and effectiveness (*r* = 0.232, *p* = 0.009). This suggests that providing choice could improve the effectiveness of group work.

#### Verbatim quotes

The verbatim quotes that support the student’s preference for choosing their group members are as follows:

‘I have no problem with group works as long as I get to choose my group members. My experience was not bad but not good either, you get people who get sick on the day of presentation and late comers.’ (Participant 91, Student, 19–35 years)

‘Through assignments, where we were given a topic and group members could be chosen on our own, except for certain subjects where the lecturer chose the groups.’ (Participant 94, Student, 19–35 years)

‘I don’t enjoy working in a group, especially 1 allocated to me.’ (Participant 107, Student, 19–35 years)

‘It is not easy to work with people of different personalities. When given the chance to choose my group members I realised that is very productive, communication is not difficult, we understand one another.’ (Participant 88, Student, 19–35 years)

‘Sometimes group work is horrible especially if we could not choose our members people tend to be lazy and some end up doing all the work.’ (Participant 37, Student, 19–35 years)

‘I have found group work to be pleasant only when given the opportunity to choose a group I know who is dedicated and hardworking therefore we choose to work with dedicated individuals we are not.’ (Participant 48, Student, 19–35 years)

### Diversity

The Mann–Whitney test for comparing enjoyment of group work and preference to do less group projects indicates that these variables significantly impact on the facilitation of group work with diversity. These results indicate that the participants who enjoy group work rated diversity higher than participants who did not enjoy group work (*p* = 0.013). The participants who preferred to do less group work rated diversity lower (*p* = 0.047), and those who agreed that the lecturer facilitated effective group work also rated diversity lower (*p* = 0.005). The Pearson’s correlation coefficient was calculated, and the results show a small correlation between the factor of diversity and participants’ overall perception of the effectiveness of their group work experience (*r* = 0.357, *p* = 0.000). The positive correlation observed indicates that a rating of diversity results in a higher rating of effectiveness.

#### Verbatim quotes

The verbatim quotes demonstrate challenges with diversity in groups:

‘During group work activity other prefers to work with people they know or people from the same corner but other struggles with race issues and tribal on national affiliation due to language and behaviour.’ (Participant 56, Student, 19–35 years)

‘Sometimes I find it racist when working with other racial people because my participation and contribution they dont take it seriously sometimes.’ (Participant 4, Student, 19–35 years)

### Overall effectiveness of group work

The Mann–Whitney test for comparing enjoyment of group work and preference for less group projects indicates that these variables significantly impact on the facilitation of group work with effectiveness. These results indicate that male participants rated effectiveness higher than female participants (*p* = 0.013). The participants who enjoy group work rated effectiveness higher than participants who did not enjoy group work (*p* = 0.000). In contrast, the participants who prefer less group projects rated effectiveness lower than participants who did not think there should be less group work (*p* = 0.000). Lastly, the participants who agreed that the lecturer facilitated effective group work rated effectiveness higher than the participants who disagreed (*p* = 0.000).

## Discussion

Graduate diagnostic radiography students are expected to display teamwork skills in the clinical setting. They are required to have knowledge about interactions between interprofessional healthcare teams and how it affects the quality of health outcomes. Hence, they should be exposed to effective group work to facilitate teamwork skills. The findings show that a majority (*n* = 81; 66.9%) of participants did not enjoy group work, as 101 (81.5%) of them preferred lesser group projects. Interestingly, 101 (81.5%) participants agreed that educators facilitated group work effectively, and 94% agreed that lecturers provided the expected outcome of group work. However, the verbatim quotes and the rating of factors contributing to effectiveness of group work contradicted these results. It appears that students were providing socially acceptable answers on the Likert scale and expressed themselves differently when given the opportunity to explain further in the open-ended questions.

### Co-operation

Student radiographers, as part of their training, are expected to understand their role in the healthcare environment and their professional responsibilities in clinical practice (Hazell et al. 2020). Teamwork is one of the skills required; hence, it was important to establish if radiography students were exposed to effective groupwork that fosters these skills before they could qualify as radiographers. The current study shows that students rated *co-operation* as ‘neutral’ on the five-point Likert scale. This result was disappointing as the students were expected to have rated co-operation high as they are almost ready to undertake community service where co-operation is needed. The participants in this study lacked the ability to work together, as group members were ‘only concentrating on their part of the work’ during group work. This was not supported by Situmorang’s (2021) study, which shows that group work taught students to solve problems collaboratively and to negotiate with members of the group. Cartwright et al. (2021) demonstrated that students had positive working relationships with group members; they were also collaborating to solve problems and were respectful of each other’s ideas. The radiography curriculum clearly prescribes that radiography students at the diploma level who exit the programme should be able to work effectively with others in the healthcare team (South African Qualifications Authority 2001). Gravett and Geyser (2014) indicate that co-operation is facilitated by an educator who actively monitors the group work process and who intervenes when conflict arises between group members. Furthermore, educators play a vital role in assisting group members in establishing mutual roles and in assigning clear roles to individual members (Gravett & Geyser 2014). The neutral ranking of co-operation in this study may imply that educators did not facilitate co-operation successfully in the universities concerned. Lundvall et al. (2021) indicated student radiographers struggled to be part of the team during their clinical placement in the training facilities. Student feedback surveys could also be employed by the radiography department to afford students the opportunity to reflect on their experience learning within the curriculum and to address any gaps. The aim of the feedback is to check whether there is constructive alignment between the curriculum and the teaching and learning of students for a review of the curriculum.

### Leadership

*Leadership* is another contributing factor identified as a structural component of effective groupwork. This study showed a moderate correlation between leadership and the effectiveness of group work implying that having a group leader could promote effective group work. These results align with Toseland and Rivas’ (2021) and Heinemann and Zeiss’ (2012) argument that for group work to be effective, a strong leader would need to co-ordinate the activities of the group. An effective leader would enable the group to meet the group goals and satisfy the social and emotional needs of the group members. However, students in this study rated leadership as ‘neutral’, with results indicating that students were dissatisfied with the selection criteria of the leadership roles and domination of certain members in the group. Therefore, leadership should be incorporated into group activities. It is recommended that before group work is assigned to students, there should be group rules to guide the group work activity. The rules can be used to prescribe the selection process leadership role in a group. This process may ensure that members are democratically elected into group roles without coercion or being dominated by other members.

### Choice

The students in this study rated *choice* as ‘neutral’ on the five-point Likert scale. These results are surprising as students generally would prefer to choose their own group members. Hall et al.’s (2022) findings show that students prefer to form their own groups even though putting them together randomly is viewed as a fair practice by educators. Thom (2020) indicates that allowing students to self-select their own groups is superior to other methods, pointing out that individuals will perform to their potential as they are more accountable to their friends. The study by Acar-Erdol and Ongoren (2020) emphasises that student satisfaction with group work is higher when students choose their own groups. In their study, under the heading ‘Structuring Process of Group Work’, the reasons that were deemed as decisive in the selection of a groupmate were ‘being close friends, getting along well, sincerity and trust’. The results show that there is a correlation between choice and the effectiveness of group work. These findings could imply that participants were not decisive in choosing the members of groups and were not satisfied with group work. Hall et al. (2022) are of the view that educators should be orientated to handle group work more effectively by guiding students in assigning roles and explaining how groups function.

### Diversity

The results showed a positive correlation between *diversity* and the effectiveness of group work, implying that diverse groups could promote the effectiveness of group work.

Current literature suggests that diversity could positively impact on group work activities (Hall et al. 2022; Samadi et al. 2022). Samadi et al. (2022) point out that there is a positive association between task-related diversity in groups and performance, and that diversity leads to positive outcomes such as innovative and creative ideas. Smaller correlations could imply that educators at the universities concerned did not take diversity into account when facilitating group work.

### Limitations

There was a paucity of literature on student radiographers’ experiences in group work; this presented a challenge as no comparisons could be made with the existing research findings. Literature was therefore based on findings from other healthcare disciplines. The study was confined to diagnostic radiography students and did not include all radiography students. The study was confined to only three universities, and therefore the results cannot be generalised.

### Recommendation for future research

For future research, it is recommended that a qualitative study be conducted for more in-depth responses from the students. The future study should include the radiography educators, as their requirements to facilitate effective group work are not known.

## Conclusion

This study was conducted to ascertain whether the final-year radiography students of the National Diploma curriculum enrolled in the diploma programme were exposed to effective group work. This study was warranted as the universities were moving away from the diploma towards a bachelor’s degree. This was driven by the change and the demand in the health sector and to align radiography qualifications with the new HEQSF. The diploma and bachelor’s degree are taught by the same educators. Thus, it was important to conduct the study to consider the interventions that could be made by the same educators to promote effective group work in the degree programme. The literature indicated that for group work to be effective, it should be structured, meaning that factors such as co-operation, leadership, choice and diversity should be incorporated into group work activities. A group activity should include a rubric to facilitate these factors, accompanied by criteria that could guide students before working as a group and how the group should interact. This could equip students with the skills required in the world of work as they foster communication and teamwork skills. The findings show that these factors were all rated ‘neutral’ on the Likert scale, suggesting that the participants were not exposed to effective group work. The results suggest that this cohort lacked communication skills and teamwork skills. It is recommended that the guidelines to facilitate effective group work in radiography education should be developed to equip educators with the knowledge to facilitate effective group work.
